# The Manners of Nations

**Published:** 1881-11

**Authors:** 


### THE MANNERS OF NATIONS.

The American claims, instinctively and implicitly, a natural and inborn right to the companionship, and, to a certain extent, the sympathy of whomsoever he meets. This is his first claim upon society at large. The Englishman claims, as instinctively and implicitly, a right to be left alone whenever and wherever he please. The one is ever aggressive, the other ever on the defensive. When the two meet on neutral ground it is not unnatural that they should tread upon each other's toes a little, metaphorically speaking. Go up to an Englishman in the street, and bluntly ask him the way to such and such a building, and he will reward the unprovoked onslaught upon his privacy with the very coolest of astonished stares. But if you preface your question with an "I beg your pardon" (acknowledging thereby that you are asking a gratuitous favor), he will answer you very civilly, indeed, and even go out of his way to show you yours. In the dense crowd coming out of one of the concerts of a Birmingham festival a well-dressed American looked into the face of an equally well-dressed Englishman (a total stranger to him) and said, jokingly, "Pretty tight squeeze, isn't it?" The Englishman replied, with withering coolness, "I'm sure I can't see why you should address me in this disgustingly familiar manner." A conversation abruptly launched into in an English railway carriage will probably be cut short at the outset by what strikes us Americans as very gratuitously rude curtness. But if the conuersation is introduced by that unfailing open sesame, "I beg your pardon," it will flow on very pleasantly, and likely as not end in an invitation to pass a week or so at a country house. Americans are often shocked at the apparently insolent bearing of German shopkeepers, especially of German bankers. It rarely occurs to them that they themselves have opened hostilities, as it were, by breaking the very first rule of the German good breeding in keeping their hats on. In Germany a man just as scrupulously takes off his hat, and keeps it off, in a shop or counting room as in a lady's parlor.—*Boston Transcript.*

---

MANY persons iron towels, fold them and place them away before they are thoroughly dry. This is an error; and sometimes leads to results not expected. In this damp condition there is a mold which forms on them called "oidium," one variety of which causes a skin disease known as ringworm.

